# Silencing of the hTERT gene through RNA interference induces apoptosis via Bax/Bcl-2 in human glioma cells

**DOI:** 10.3892/or.2012.1952

**Published:** 2012-08-06

**Authors:** TUO WANG, YAN XUE, MAODE WANG, QIANG SUN

**Affiliations:** 1 Department of Neurosurgery, The First Affiliated Hospital, Xi'an Jiaotong University, Xi'an 710061; 2 Department of Pharmacology, School of Medicine, Xi'an Jiaotong University, Xi'an 710061, P.R. China

**Keywords:** telomerase, hTERT, glioma, apoptosis

## Abstract

Glioma cells are characterized by their invasiveness and resistance to conventional therapeutics. The downregulation of human telomerase reverse transcriptase (hTERT) can lead to decreased cell proliferation and/or the induction of apoptotic cell death in cancer cells but has rarely been reported in glioma cells. Here, we assessed the effect of the silencing of the hTERT gene on cell apoptosis and its possible molecular mechanism in T98G glioma cells. We found that the silencing of the hTERT gene in T98G cells significantly decreased cell proliferation and telomerase activity, increased the number of cells in G1 phase and decreased the number of cells in S phase, and induced apoptosis via decreasing the protein level of Bcl-2 and c-Myc and increasing the protein levels of Bax and p53.

## Introduction

Gliomas, which originate from the predominant glial tissue in the central nervous system, are the most common types of malignant brain tumors in adults. Patients suffering from malignant gliomas have a life-span between 9 and 12 months after the diagnosis of grade IV and 2 years after the diagnosis of grade III gliomas (1–4). More than 90% of cancers show activated telomerase, including gliomas. Telomerase activity permits cancer cell immortalization and promotes tumorigenesis. The expression of telomerase and its genetic variation has been correlated with malignant glioma progression (5) and is therefore an important enzyme to target for improving the prognosis and treatment of gliomas (6). Three components of human telomerase have been identified: the RNA component (hTER) (7), the telomerase-associated protein (TEP1) (8,9), and the telomerase reverse transcriptase (hTERT) (6,10,11). Although both hTER and hTERT are necessary for telomerase activity, hTERT is the major determinant of telomerase activity. hTERT, the catalytic subunit of telomerase, is the rate-limiting step in the activation of telomerase and is correlated with the pathological grade and type of human glioma.

Apoptosis plays a key role in the pathogenesis of cancers, and the genes relating to this process are of interest in studies on cancer onset and progression. Glioblastomas pose a challenge in neuro-oncology because of their resistance to apoptosis and conventional therapies (12,13). Bax and Bcl-2 are transcriptional targets of the tumor suppression protein p53, which is responsible for the induction of cell cycle arrest and/or apoptosis in response to DNA damage. The progression of cancer mainly depends on the balance between pro-apoptotic proteins, such as Bax, and anti-apoptotic proteins, such as Bcl-2 (14). The inhibition of hTERT rapidly induces apoptosis in gastric cancer, but this effect has rarely been reported in gliomas.

In this study, we used siRNA to downregulate hTERT in T98G cells and investigated the effect of hTERT on T98G cell proliferation, apoptosis and cell cycle progression. We also explored its possible molecular mechanism.

## Materials and methods

### Cell culture

The human glioblastoma cell line T98G was procured from the Huaxi Medical Center (Sichuan University, Chengdu, Sichuan, China). T98G was derived from a human glioblastoma multiforme tumor. We propagated T98G cells in DMEM (Gibco-BRL, Carlsbad, CA, USA) supplemented with 10% fetal bovine serum (Invitrogen, Carlsbad, CA, USA) and antibiotics in a humidified incubator containing 5% CO_2_ at 37°C.

### Small-interfering RNA design and transfection

The cDNA sequence of the human telomerase reverse transcriptase gene (hTERT) (GenBank accession number NM_198253) was used to design small-interfering RNA (siRNA). The specificity of the siRNA sequence was confirmed through BLAST searches, and the sequence did not show any homology to other known human genes. A random coding sequence of siRNA was used as a negative control. The specific small-interfering RNAs (siRNAs) were synthetized and sequenced by Shanghai GenePharma Co., Ltd. (Shanghai, China). For hTERT, the siRNA sense sequence was 5′-CGGUGUACGCCGAGACCA ATT-3′, and the anti-sense sequence was 5′-UUGGUCUCGG CGUACACCGGG-3′. The negative-control siRNA sense sequence was 5′-UUCUCCGAACGUGUCACGUTT-3′, and the anti-sense sequence was 5′-ACGUGACACGUUCGGA GAATT-3′. The most effective construct was selected based on the percentage knockdown of hTERT at both the mRNA and protein levels. For transfection, 2×10^5^ cells were seeded into each well of a 6-well tissue culture plate (Costar). The next day (when the cells were 70–80% confluent), the culture medium was aspirated, and the cell monolayer was washed with pre-warmed sterile phosphate-buffered saline (PBS). The cells were transfected with Lipofectamine 2000 reagent (Invitrogen) in accordance with the manufacturer's protocol. The cells were continuously cultured until they were harvested for analysis. The transfection efficiency was monitored with the expression of carboxyfluorescein (FAM) under a phase-contrast fluorescent microscope (Olympus IX71, Japan).

### Reverse transcription-polymerase chain reaction (RT-PCR) and western blotting to examine the hTERT mRNA and protein levels

RT-PCR and western blotting were performed to examine the downregulation of hTERT mRNA and protein levels, respectively, after the knockdown of hTERT. The following primer sequences were used for the PCR amplification of hTERT: sense strand, 5′-ATGGCTGCGTGGTGAAC TTG-3′, and antisense strand, 5′-AGGTGAGACTGGCTCT GATGG-3′. The primers were used to amplify 1,000 ng of total RNA with a single-step RT-PCR kit (Invitrogen) with a PCR cycler (Eppendorf, Westbury, NY, USA) at an annealing temperature of 56°C. Western blotting was performed with an hTERT antibody (Santa Cruz Biotechnology, Santa Cruz, CA, USA) to determine hTERT protein levels as described below. Both RT-PCR and western blotting images were quantified using Gel-Pro Analyzer software (Media Cybernetics, Silver Spring, MD, USA).

### Flow cytometry cell cycle analysis

T98G cells (1×10^7^) were cultured in each well of a 6-well plate to 70–80% confluence with normal culture medium. The cell were treated with hTERT siRNA (100 nM) for 2 or 3 days, trypsinized, and stained with propidium iodide with the Cellular DNA Flow Cytometric Analysis Reagent Set (Boehringer Mannheim, Indianapolis, IN, USA). The cells were harvested and fixed with 3 ml of ice-cold 70% ethanol overnight. Then, the cells were incubated with RNase A (1 mg/ml; Sigma, St. Louis, MO, USA) for 10 min at room temperature. The DNA was stained with propidium iodide (50 μg/ml) for at least 1 h at 4°C, and the DNA content was determined with flow cytometry (Beckman Coulter, San Diego, CA, USA). The data were analyzed with CellQuest software (Becton-Dickinson, San Jose, CA, USA).

### Telomerase activity (TRAP) assay

Telomerase activity was determined with a PCR-based telomeric repeat amplification protocol (TRAP) enzyme-linked immunosorbent assay (ELISA) kit (Roche, Mannheim, Germany) according to the manufacturer's protocol. In brief, T98G cells were collected 48 h after siRNA transfection. The cells were washed three times with cold PBS, homogenized in 200 μl cell lysis buffer, and incubated on ice for 30 min. For the TRAP reaction, 2 μl of cell extract was added to 25 μl of reaction mixture, and sterile water was added to a final volume of 50 μl. PCR was then performed as follows: primer elongation (20 min, 25°C), telomerase inactivation (5 min, 94°C), product amplification for 30 cycles (94°C for 30 sec, 50°C for 30 sec, and 72°C for 90 sec) and then balance (10 min at 72°C). A total of 5 μl of PCR products was added to a streptavidin-coated 96-well plate and hybridized to a digoxigenin (DIG)-labeled telomeric repeat-specific detection probe. The immobilized PCR products were detected with peroxidise-conjugated anti-DIG antibody. After addition of the stop reagent, the plate was assessed with a plate reader at a wavelength of 450 nm within 30 min.

### MTT assay

T98G cells were incubated in 96-well plates, with each well containing 200 μl of medium. The cells were divided into the three following groups: i) blank group, ii) control siRNA group, and ii) hTERT siRNA group. The transfection of siRNAs was performed the following day, as previously described (15). The rate of cellular proliferation was measured every 24 h for 120 h. At the end of each time point, 20 μl of 5 mg/ml MTT (Sigma) was added to each well. Four hours later, 200 μl of DMSO was added to the MTT-treated wells, and the absorption at 492 nm was determined with a spectrometer. Each experimental condition was performed in triplicate.

### Apoptosis assay

Flow cytometry assays and TUNEL assays were performed to detect cell apoptosis. A total of 1×10^6^ cells were transfected with siRNA. At 30 h post-transfection, the cells were harvested, washed twice with PBS, and resuspended in 200 μl Annexin V binding buffer (10 mM HEPES, 140 mM NaCl, 2 mM MgCl_2_, 5 mM KCl, and 2.5 mM CaCl_2_, pH 7.4). A total of 10 μl FITC-conjugated Annexin V (Beijing Biosea Biotechnology Co., Beijing, China) was added according to the manufacturer's protocol. After incubation for 20 min at room temperature in the dark, another 400 μl of binding buffer was added, and the samples were immediately analyzed using FACSCalibur. In total, 1×10^4^ cells were collected and analyzed with CellQuest software. Apoptotic cells are expressed as a percentage of total cells.

The TUNEL assay was performed to detect apoptosis as a marker of cell death. Briefly, T98G cells were fixed with 4% paraformaldehyde for 10 min and then incubated for 60 min with TdT. Streptavidin-HRP was added to the samples, which were then incubated in the dark for 30 min and incubated for 10 min with DAB. Positively stained cells were visualized and photographed using a microscope.

### Western blotting for molecules involved in cell apoptosis regulation in vitro

Cells were lysed in M-PER Mammalian Protein Extraction Reagent (Pierce Biotechnology, Inc., Rockford, IL, USA) supplemented with a protease inhibitor cocktail (Roche, Indianapolis, IN, USA) followed by centrifugation at 12,000 rpm for 10 min. After centrifugation, the cell lysates were collected, and the protein concentrations of the cell lysates were measured. Proteins (10–20 μg) were resolved through SDS-PAGE and then transferred to PVDF membranes (Bio-Rad Laboratories, Hercules, CA, USA). The blots were incubated with primary antibodies in 3% BSA/TBST at 4°C overnight followed by incubation with secondary antibodies at room temperature for 1 h. The protein signals were detected with the ECL method (16).

### Statistical analysis

The mean and standard deviation (SD) were calculated for all of the quantitative data. The results were statistically evaluated using a one-way analysis of variance (ANOVA). The least significant difference method was used to compare the mean values of control or negative control siRNA-treated groups with hTERT siRNA-treated groups. A value of P<0.05 was considered statistically significant.

## Results

### Downregulation of hTERT mRNA and protein levels in T98G cells

We initially tested three sets of siRNAs for hTERT knockdown. The preliminary results showed one set being particular effective (data not shown); thus, all of the experiments described in this report were performed with this siRNA (for the sequence, see the methods section).

**Figure 1:**
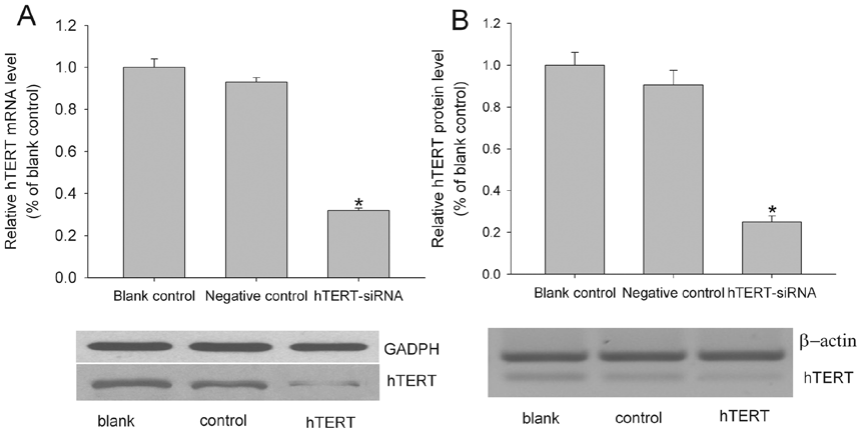
The transfection of hTERT siRNA inhibits hTERT expression at both the mRNA and protein level. (A) RT-PCR results for hTERT mRNA expression. The values indicate the mean ± SD of 3 independent experiments in each group (^*^P<0.01 compared to the control mean values). (B) Western blotting of hTERT protein levels. The data are representative of 3 independent experiments (^*^P<0.01 compared to the control mean values).

The hTERT siRNA was transiently transfected into the T98G glioma cell line. After 48 h, the hTERT mRNA and protein levels were quantified with real-time RT-PCR and western blotting, respectively. As shown in Fig. 1A, hTERT siRNA transfection significantly reduced the amount of hTERT mRNA. The level of hTERT mRNA in the hTERT siRNA-treated group was ~40% of the blank group. The control siRNA had no effect on the hTERT mRNA level. The hTERT siRNA was also successful in knocking down hTERT protein expression. As shown in Fig. 1B, although the β-actin internal control showed equal loading among the three groups, the level of hTERT protein was noticeably lower in the hTERT siRNA-treated group compared to both the blank and the negative siRNA-treated groups, suggesting that the hTERT siRNA treatment could effectively reduce the hTERT protein level.

### Downregulation of telomerase activity in brain glioma cells after hTERT siRNA transfection

**Figure 2:**
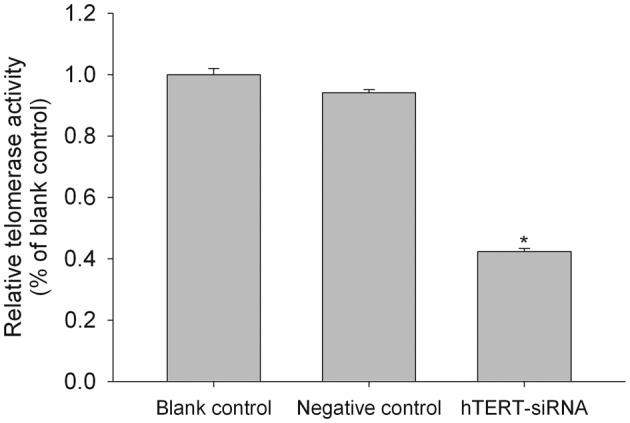
Telomerase activity levels after transfection with hTERT siRNA. Telomerase activity levels quantified with TRAP assays in T98G cells at 48 h after a 6-h exposure to hTERT siRNA or control siRNA and in untransfected cells. The data are shown as the mean ± SD (error bar) of 3 experiments (^*^P<0.01 compared to the control mean values).

The level of telomerase activity in human gliomas has been shown to correlate significantly with hTERT mRNA expression (17). Consistent with this result, we found that the hTERT siRNA-transfected cells showed a 42% reduction in telomerase activity, as determined with a PCR-based telomeric repeat amplification protocol (TRAP) ELISA (Fig. 2).

### hTERT siRNA inhibited cell viability in vitro

**Figure 3:**
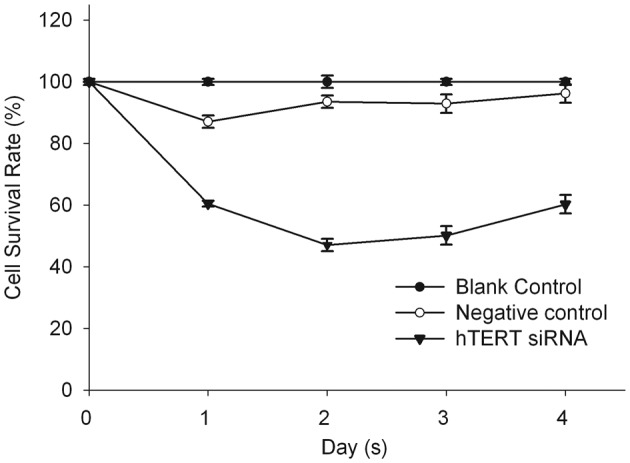
The determination of the cell viability of T98G cells after transfection with hTERT siRNA. MTT assay for the determination of cell viability. The data are the mean ± SD of 6 independent experiments.

Decreased telomerase activity is associated with arrested cell growth; therefore, we determined whether the hTERT siRNA-induced reduction in telomerase activity affected the cell viability of the T98G cell line. The cells were transfected with hTERT siRNA, and the number of viable cells was determined with the MTT assay every 24 h for 4 days. As shown in Fig. 3, hTERT siRNA significantly decreased the percentage of viable cells in the T98G cell line. The decrease was rapid: only ~57.7% of cells were viable after 24 h, and only 47% of cells survived after 48 h. However, the inhibitory effect of the siRNA was temporary, and the T98G cells began proliferating 3 days after the exposure to siRNA.

### The effect of hTERT siRNA on the cell cycle

**Figure 4:**
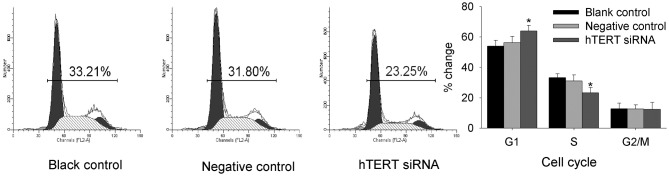
Cell cycle analysis after transfection with hTERT siRNA for 48 h. (A) FACS histograms of T98G cells showing cell cycle arrest. The cells were treated with 50 μg/ml propidium iodide for 20 min at 4°C in the dark prior to cell cycle analysis. An increase in the cell population in G1 phase indicated cell cycle arrest. (B) Quantitative representation of FACS data of the population of cells in G1, S and G2/M phase. The data are representative of 4 independent experiments (^*^P<0.01 compared with the mean values of control siRNA-treated cells).

To determine whether hTERT siRNA affected the cell cycle of malignant glioma cells, flow cytometry was performed. As shown in Fig. 4, the flow cytometry assay showed the after transfection with hTERT siRNA, the number of cells in G1 phase was increased, but the number of cells in S phase was decreased. There was no alteration in the cell population in subG1, S, or G2/M phase in the negative-control group.

### The decrease in cell viability caused by hTERT siRNA is due to an increase in apoptosis

**Figure 5:**
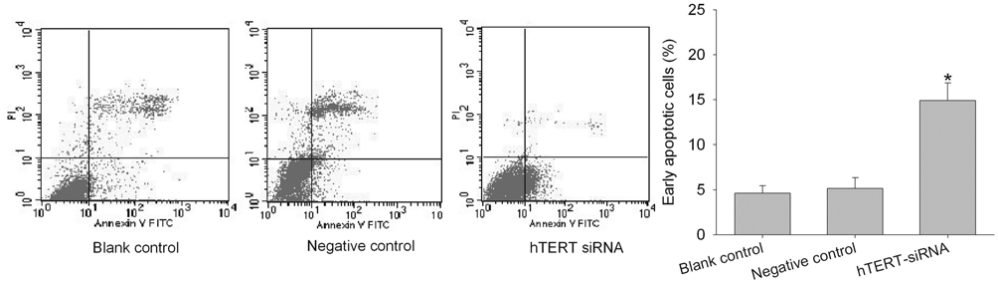
Flow cytometry analysis of early cell apoptosis after hTERT siRNA. T98G cells were transfected with hTERT siRNA. At 48 h post-transfection, the percentage of cell apoptosis was analyzed with flow cytometry with Annexin V-FITC. The M1 numbers indicate the corresponding proportions of apoptotic cells that display positive Annexin V-FITC labeling. The data indicate that hTERT siRNA significantly promotes T98G cell early apoptosis. Columns representing the flow cytometry data are presented on the right. The data are means ± SD of values from three independent experiments. ^*^P<0.05 compared with the mean values of control siRNA-treated cells.

**Figure 6:**
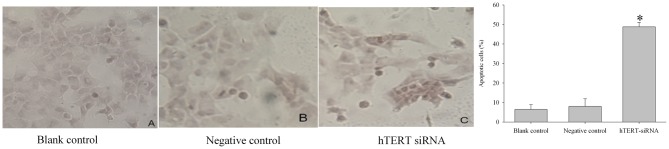
Effect of hTERT-siRNA on the apoptosis of brain cancer T98G cells. The apoptosis of brain cancer cells induced by hTERT siRNA. Apoptotic cells were detected with a TUNEL assay. The columns representing the TUNEL data are presented at left. The data shown indicate that hTERT siRNA significantly promotes T98G cell apoptosis. The experiments were performed in triplicate, with similar results (^*^P<0.01 compared with the mean values of control siRNA-treated cells).

To determine whether the decrease in cell viability caused by the hTERT siRNA was due to an increase in apoptosis, we determined the number of early apoptotic cells in the untransfected, negative control- and hTERT siRNA-transfected cells with Annexin V-FITC and propidium iodide (PI) labeling followed by fluorescence-activated cell sorting (FACS). As shown in Fig. 5, 48 h after siRNA transfection, the number of early apoptotic cells was increased significantly. Cell apoptosis was then examined using a TUNEL assay. hTERT siRNA dramatically increased the number of cells that stained positive for TUNEL within the nucleus (Fig. 6), indicating a high incidence of apoptosis.

### hTERT siRNA downregulates the molecules involved in apoptosis

**Figure 7:**
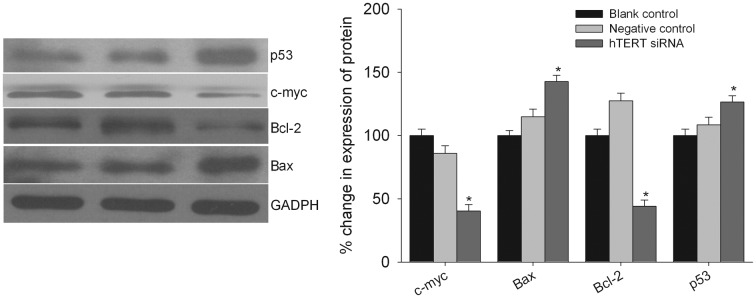
Effect of hTERT-siRNA on some molecules involve in cell apoptosis. (A) Western blotting of p53, c-Myc, Bcl-2 and Bax protein level. The GAPDH was used as an internal control. (B) Columns representing the western blotting data are presented on the right. The data are the mean ± SD of 6 samples. (^*^P<0.01 compared with the mean values of control siRNA-treated samples).

To confirm the molecular mechanism of the inhibition of cell apoptosis after the downregulation of hTERT, we determined the protein levels of the important molecules involved in this process (Fig. 7). A western blotting assay showed that the levels of several proteins involved in the apoptotic pathway were different. The expression level of Bcl-2 and c-Myc was decreased whereas the expression level of Bax and p53 was increased after the treatment of T98G cells with hTERT siRNA (Fig. 7).

## Discussion

This study shows that in T98G glioma cells, siRNAs targeting the hTERT gene can be efficiently delivered and results in the rapid inhibition of telomerase activity and cell growth. The inhibition of cell growth is associated with cell cycle arrest and the promotion of cell apoptosis through transcriptional and/or translational upregulation and/or downregulation of the molecules involved in this process.

RNA interference has emerged as an effective method for the specific inhibition of gene expression both *in vitro* and *in vivo*. Telomerase plays a key role in cellular immortality and tumorigenesis. Telomerase is a distinctive candidate for the targeted gene therapy of malignant gliomas because the vast majority of malignant gliomas express telomerase activity, whereas normal brain tissues do not (18–20). Telomerase and its major catalytic subunit hTERT are upregulated in most cancers, including glioblastomas (17,21). Moreover, hTERT expression has been correlated with poor survival in glioblastoma patients (22).

Previous studies have demonstrated that the downregulation of hTERT in glioblastoma cells is correlated with a decrease in cell viability, proliferation, tumor cell migration, and invasion through the downregulation of the molecules involved in these processes and cell cycle inhibition (17,21). In the present study, siRNA directed against hTERT resulted in >70% suppression of hTERT at the mRNA and protein levels. Furthermore, siRNA targeting hTERT significantly inhibited cell proliferation and increased apoptosis by downregulating hTERT expression and decreasing telomerase activity in T98G human glioma cells.

In cancer cells, the stabilization of telomeres through the reactivation of telomerase has been suggested to be a crucial step during cellular immortalization and tumorigenesis. Moreover, telomerase inhibition is associated with the induction of apoptosis and senescence. Earlier studies have shown that the selective silencing of hTERT using hTERT siRNA and oligonucleotides targeting the RNA component of telomerase induces both apoptosis and senescence in Barrett's adenocarcinoma cells (5,18). In our present study, silencing hTERT using hTERT siRNA induced apoptosis in T98G glioma cells.

c-Myc contributes to apoptosis via its interaction with a number of apoptotic pathways. Pathways involving p53 and Bax (Bcl-2-associated × protein) have been shown to be activated by c-Myc (6). In addition, Bcl-2 suppresses c-Myc-induced apoptosis without affecting the ability of c-Myc to regulate the progression of the cell cycle from G1 phase to S phase. c-Myc-induced tumorigenesis is the result of the suppression of apoptosis by cooperating oncogenes and the activation of S phase by c-Myc, leading to cell proliferation (23,24). siRNA-mediated c-Myc downregulation resulted in an inhibition of cellular proliferation and clonogenic growth, the inhibition of G1-S phase cell cycle progression, and a decrease in human telomerase reverse transcriptase (hTERT) expression and telomerase activity in human medulloblastoma cells (25).

Anti-apoptotic Bcl-2 family members are highly overexpressed in malignant gliomas. The induction of apoptosis by downregulating hTERT expression and decreasing telomerase activity was shown in changes in the expression levels of proteins responsible for the regulation of apoptosis. Bax and Bcl-2 are the two principal genes involved in the regulation of apoptosis. Previous studies have demonstrated that during apoptosis induction, bax protein levels are upregulated, which has a well-known pro-apoptotic effect, Bcl-2, which protects cells from apoptosis, is downregulated. According to our results, the anticancer cell growth inhibition is due to the deregulation of apoptosis induction.

The p53 tumor suppressor is another cell cycle regulator that is frequently altered in brain tumors. During cell DNA damage or cytotoxic stress, there is an increase in p53 protein levels that induces cell growth arrest, DNA repair mechanisms, and apoptosis (26–28).

In conclusion, our study demonstrated that the knockdown of hTERT effectively inhibited the cell viability of human glioblastoma cells by increasing the positive index of apoptotic cells via decreasing the expression of Bcl-2 and c-Myc and cell cycle arrest at G0/G1 phase. Therefore, hTERT siRNA offers a potential therapeutic regimen for effectively controlling the growth of human glioblastoma cells.
